# Distinct Roles of Smooth Muscle and Non-muscle Myosin Light Chain-Mediated Smooth Muscle Contraction

**DOI:** 10.3389/fphys.2020.593966

**Published:** 2020-12-03

**Authors:** Jie Sun, Yan-Ning Qiao, Tao Tao, Wei Zhao, Li-Sha Wei, Ye-Qiong Li, Wei Wang, Ye Wang, Yu-Wei Zhou, Yan-Yan Zheng, Xin Chen, Hong-Chun Pan, Xue-Na Zhang, Min-Sheng Zhu

**Affiliations:** ^1^Model Animal Research Center, School of Medicine, Nanjing University, Nanjing, China; ^2^Key Laboratory of MOE for Modern Teaching Technology, Shaanxi Normal University, Xi’an, China; ^3^College of Life Sciences, Anhui Normal University, Wuhu, China

**Keywords:** smooth muscle, contraction, myosin, MYL9, MLCK, intestine, bladder, uterus

## Abstract

Both smooth muscle (SM) and non-muscle (NM) myosin II are expressed in hollow organs such as the bladder and uterus, but their respective roles in contraction and corresponding physiological functions remain to be determined. In this report, we assessed their roles by analyzing mice deficient of *Myl9*, a gene encoding the SM myosin regulatory light chain (SM RLC). We find that global *Myl9*-deficient bladders contracted with an apparent sustained phase, despite no initial phase. This sustained contraction was mediated by NM myosin RLC (NM RLC) phosphorylation by myosin light chain kinase (MLCK). NM myosin II was expressed abundantly in the uterus and young mice bladders, of which the force was accordingly sensitive to NM myosin inhibition. Our findings reveal distinct roles of SM RLC and NM RLC in SM contraction.

## Introduction

Smooth muscle (SM) cells line the walls of hollow organs, including the gastrointestinal tract, vasculature, uterus and bladder, and they maintain homeostasis and responses to imposed signals (Somlyo and Somlyo, 1994). SM contraction is initiated by crossbridge movement of SM myosin II-based thick and F-actin-based thin filaments. During this process, myosin activity is significantly stimulated by regulatory light chain (RLC) phosphorylation when in contact with actin (Rosenfeld et al., 1998). Therefore, SM RLC phosphorylation by calcium/calmodulin-dependent myosin light chain kinase (MLCK) is fundamentally required for SM contraction and hollow organ physiological activities (Kamm and Stull, 1985; Somlyo and Somlyo, 2003; He et al., 2008, 2011, 2013; Qiao et al., 2014). Interestingly, there are studies showing that SM potentially produces force in an SM myosin-independent manner (Morano, 2003; Gunst and Zhang, 2008), but the underlying mechanism remains to be determined. Non-muscle (NM) myosin II is highly similar to SM myosin II based on the evidence that both their heavy chains (MHCs) and RLCs are phylogenetic closely related to each other (Grant et al., 1990; Berg et al., 2001), and both their activation need RLC phosphorylation (Sellers, 2000). Moreover, the fractional content of NM MHCs was reported to significantly decrease with the development in various SM tissues of mouse (Gaylinn et al., 1989; Eddinger and Wolf, 1993; Eddinger and Meer, 2007). Thus, we hypothesize that NM myosin II might be involved in force production.

Myosin II is one of the most important superfamilies of motor proteins, consisting of six subunits, including two MHCs, two RLCs, and two essential light chains (ELCs; Craig and Woodhead, 2006). SM expresses two primary myosin isoforms, SM myosin and NM myosin, and SM myosin is much more abundant (Morano, 2003; Eddinger and Meer, 2007). *Myl12a* and *Myl12b* encode NM RLCs, *Myl9* encodes SM RLC (Park et al., 2011), *Myh9* and *Myh10* encode NM MHC, and *Myh11* encodes SM MHC. SM RLC and NM RLC proteins share more than 90% identity in their amino acid sequences and are phosphorylated by kinases (Park et al., 2011). The phosphorylation of these RLCs leads to the activation of myosin activity necessary for multiple physiological processes (Trybus and Lowey, 1988; Seow, 2005; Milton et al., 2011). Generally, the fundamental role of NM myosin II involves cell division, cell migration, and developmental processes (Vicente-Manzanares et al., 2009; Wang et al., 2010). There are also lines of evidence pointing to a possible role of NM RLC phosphorylation in SM contraction, e.g., NM RLC in SM may be phosphorylated in response to membrane depolarization and G protein-coupled receptor agonists (Yuen et al., 2009); pharmacological inhibition of NM myosin II inhibits SM contraction (Rhee et al., 2006; Zhang et al., 2009, 2011, 2012; Chen et al., 2018); the residual force evoked by SM MHC-deficient bladders can be inhibited by an NM myosin II inhibitor (Morano et al., 2000; Löfgren et al., 2003); and NM myosin II regulates actin polymerization-integration signaling during SM contraction (Zhang and Gunst, 2017; Zhang et al., 2018). However, due to the difficulty in distinguishing SM myosin-developed force, the genesis of NM myosin II-mediated force and its physiological relevance in hollow organs is controversial. Here, we deleted the *Myl9* gene in mice to inhibit the force produced by SM myosin II. The global deletion of *Myl9* abolished the robust contraction of SMs and induced profound functional impairments in hollow organs, revealing the fundamental role of SM myosin II in SM contractility. In mutant bladder SM, however, the sustained force was even greater than that in the control (CTR). This observation suggests the existence of SM RLC-independent contraction in the bladder. A substantial analysis demonstrated that the sustained force was mediated by NM RLC through phosphorylation by MLCK, and this NM RLC-mediated contraction was necessary for tethering fluid tension in some hollow organs, such as the bladder and uterus. As NM RLC did not functionally or biochemically compensate for SM RLC, our results revealed independent and distinct roles of SM RLC and NM RLC in SM contraction as well as hollow organ functions.

## Materials and Methods

### Animals

*Myl9* flox mice were generated by the bacterial artificial chromosome (BAC) retrieval method and ES cell targeting as previously reported (Liu et al., 2003), which was described in detail below. SMA-Cre mice (Wu et al., 2007) and CMV-Cre mice (JAX:006054, RRID:IMSR_JAX:006054, Schwenk et al., 1995) were used to generate Myl9 SM specific and global deletion mice lines. Wildtype (WT) C57BL/6 mice were also used in some indicated experiments. Embryos were generated by timed mating. The day on the appearance of vaginal plug was designated embryonic day 0.5 (E0.5), and the day on which the pups were born was designated postnatal day 0 (P0). All mice were SPF and housed in a facility in accordance with AAALAC guidelines and under an animal protocol approved by the Institutional Animal Care and Use Committee (IACUC) of Model Animal Research Center (MARC) of Nanjing University.

### *Myl9* Knockout Mice Generation

The correctly targeted ES clones were selected by long-range PCR and Southern blotting and injected into blastocysts to generate chimeric mice, which were crossed onto a C57BL/6 background. The *Myl9* floxed allele was identified by genotyping using primers: forward, 5'-TGGGCTGCCCTAGTGGTGAA-3' and reverse, 5'-AAACCTCGATGCCCCAGCCT-3', in which, the WT allele generated a PCR product of 242 bp and the floxed allele generated a PCR product of 340 bp. *Myl9*-knockout allele was identified by the same forward primer and the altered reverse primer 5'-CCATCTATGCTCAGGGCTGC-3'.

### RNA Extraction, Reverse-Transcription, and Real-Time Quantitative PCR

Total RNA was extracted from fresh isolated tissues using Trizol reagent (Takara, Kyoto, Japan) following the manufacturer’s instructions. cDNA was reverse-transcribed using the HiScript Q RT SuperMix for qPCR (+gDNA wiper) kit (Vazyme, Nanjing, China) following the manufacturer’s instructions. PCR was performed to determine the expression level of *Myl9* using the primers: forward, 5'-TCAGGCTTCATCCACGAG-3'; reverse, 5'-GGGCAGCTAAGAACAGCTTAG-3', and of *Actb* as internal control using: forward, 5'-ACTGCCGCATCCTCTTCCTC-3'; reverse, 5'-AAGCACTTGCGGTGCACGA-3'. Real-time quantitative PCR was performed using an ABI Step One Plus (ThermoFisher Scientific, MA, United States) and the AceQ qPCR SYBR Green Master Mix kit (Vazyme) following the manufacturer’s instructions. Primers used were as follows: *Myl9*, forward, 5'-CTGAACCGTAAGACCCACAG-3'; reverse, 5'-GGGGATGCTGGGAATTAGTG-3'; *Myl12a*, forward, 5'-GGGCCCTAGACTTTGTCTTT-3', reverse, 5'-AACACAGCGAAATCTGTCCTT-3'; *Myl12b*, forward, 5'-CTGCGTGCCACTTAGTTTCT-3', reverse, 5'-ACAACATTGTCCCCCGTTC-3'; *Myl6*, forward, 5'-GACCGTGGCCAAGAACAA-3', reverse, 5'-CAGCCATTCAGCACCATCCG-3'; *Myh9*, forward, 5'-CCGGCAAGGTGGACTATAAA-3', reverse, 5'-GTCTTGAAGGCACCAGGTAG-3'; *Myl10*, forward, 5'-GGGAAGAAACGCCATGAGAT-3', reverse, 5'-CGTTCAAGTTCCCCAGGAAT-3'; *Myl11*, forward, 5'-ACAGAGAGCAATGAGGCCAT-3', reverse, 5'-CCGAGCGTCCATTTCTTCTT-3'; and *Actb*, forward, 5'-CTTTGCAGCTCCTTCGTTGC-3', reverse, 5'-CCTTCTGACCCATTCCCACC-3'.

### Measurement of SM Isometric Contraction

Measurement of the isometric contraction of intestinal SM, bladder SM, uterine SM, and aortic SM was performed as described (He et al., 2008; Chen et al., 2015; del Campo and Ferrer, 2015). The forces of all SM tissues were recorded using a PowerLab device (AD instruments, NS, Australia). The LabChart 5 program was used for data acquisition and analysis.

Jejunum SM contraction: A freshly isolated 6 mm jejunum ring was mounted in a longitudinal orientation in an organ incubation chamber (PanLab, Barcelona, Spain) containing H-T buffer (137.0 mM NaCl, 2.7 mM KCl, 1.8 mM CaCl2, 1 mM MgCl2∙6H2O, 5.6 mM D-glucose, and 10 mM HEPES, pH 7.4) with bubbling O_2_ at 37°C. The resting tension was set to 0.1–0.3 g, and 87 mM KCl and 100 μM acetylcholine (ACh; Cat#A2661, Sigma-Aldrich, MO, United States) were used to induce SM contraction.

Aortic SM contraction: A 2 mm aortic ring was mounted in a myograph chamber (610-M; Danish Myo Technology, Aarhus, Denmark) containing H-T buffer with bubbling O_2_ at 37°C. The resting tension and corresponding diameter were determined by measuring the circumference-pressure curve. Then, 124 mM KCl and 10 μM U46619 (Cat#D8174, Sigma-Aldrich) were applied to induce aortic SM contraction.

Bladder SM contraction: The bladder was isolated and immersed in Krebs buffer (119.0 mM NaCl, 4.7 mM KCl, 1.17 mM MgSO_4_, 2.5 mM CaCl_2_, 20 mM NaHCO_3_, 1.18 mM KH_2_PO_4_, and 11 mM glucose). For P28 mice, the cleaned bladder SM layer was cut into 3 mm long and 1 mm wide pieces in the longitudinal direction. For E18.5 mice, the bladder SM layer was cut into 2.8–3 mm long and 1 mm wide pieces in the circular direction. One end of the SM strips was tied to a steel rod, and the other end was fixed on a force transducer. Then, they were placed into an organ incubation chamber at 37°C and bubbled with gas containing 95% O_2_ and 5% CO_2_. The resting force was set to 0.1 g, and 124 mM KCl and CCh (Cat#C4382, Sigma-Aldrich) were used to induce bladder SM contraction.

Uterine SM contraction: One side of the uterus was cut along the longitudinal axis. After carefully removing the endometrium, 1 cm long and 1 mm wide strips were cut. The strips were incubated at 37°C in Krebs buffer bubbled with gas containing 95% O_2_ and 5% CO_2_, and 60 mM KCl and 100 nM oxytocin (Cat#P1029, Selleck, TX, United States) were applied to induce contraction.

### Measurement of Myosin RLC Phosphorylation Levels

Fresh SM tissues were stimulated as described in the main text; then, they were snap-frozen in liquid nitrogen and quickly immersed into an acetone solution containing 10% trichloroacetic acid (TCA) and 10 mM DTT and stored at −80°C overnight. The samples were homogenized in a water solution containing 10% TCA and 10 mM DTT. The proteins were pelleted and subsequently washed once with acetone and twice with diethyl ether, dissolved in a urea sample buffer, and loaded onto urea/glycerol-PAGE gels. The urea/glycerol-PAGE gel can separate non-phosphorylated and phosphorylated NM RLC proteins, both of which can be detected by a single antibody to NM RLC. The separated proteins were transferred to PVDF membranes, fixed with glutaric dialdehyde, blocked, and incubated with Myl12a antibody (Cat#16287-1-AP, Proteintech, IL, United States).

### Western Blotting

To analyze protein expression in SM tissues, proteins were extracted using the TCA method as described above. The lysates were supplemented with Laemmli buffer and boiled at 95°C for 10 min; then, they were subjected to SDS-PAGE and transferred to PVDF membranes. The membranes were blocked in 5% skimmed milk and incubated with the following primary antibodies: rabbit anti-SM-MHC (Cat#ab124679, RRID:AB_10975311, Abcam, MA, United States), rabbit anti-SM RLC (Cat#ab191393, RRID:AB_2721112, Abcam), rabbit anti-NMIIA (Cat#3403, RRID:AB_2147297, Cell Signaling Technology, MA, United States), rabbit anti-NMIIB (Cat#3404, RRID:AB_1264210, Cell Signaling Technology), rabbit anti-LC_17_ (Cat#BS5807, Bioworld, MN, United States), mouse anti-*β*-actin (AC15, Cat#A5441, RRID:AB_476744, Sigma-Aldrich), and mouse anti-SMA (Clone 1A4, Cat#MS-113-P0, RRID:AB_64001ThermoFisher Scientific) at 4°C overnight. After extensive washes with Tris-buffered saline containing 0.1% Tween-20, the membranes were incubated with HRP-conjugated secondary antibodies at room temperature for 1 h. The bands were imaged using an AI600 imager (GE healthcare, WI, United States), and the densities were quantified using NIH ImageJ software (v1.48).

### H&E Staining

Fresh isolated SM tissues, including the jejunum, bladder, and aorta, were fixed in 4% paraformaldehyde, dehydrated with an ethanol series, and embedded in paraffin. The tissues were sectioned at 6 μm, dewaxed, rehydrated and stained with H&E according to standard histological procedures. Images were captured using Olympus BX53 light microscope (Olympus, Tokyo, Japan).

### Statistical Analysis

In all experiments, *n* represents the number of mice used. Data are presented as means ± SEM. The statistical analysis was performed using Prism 6.05 software. Student’s *t*-test was used as indicated in the figure legends. ^*^p < 0.05, ^**^p < 0.01, ^***^p < 0.001, and ^****^p < 0.0001.

## Results

### Establishment and Phenotypic Characterization of SM RLC-Knockout Mice

**Figure 1:**
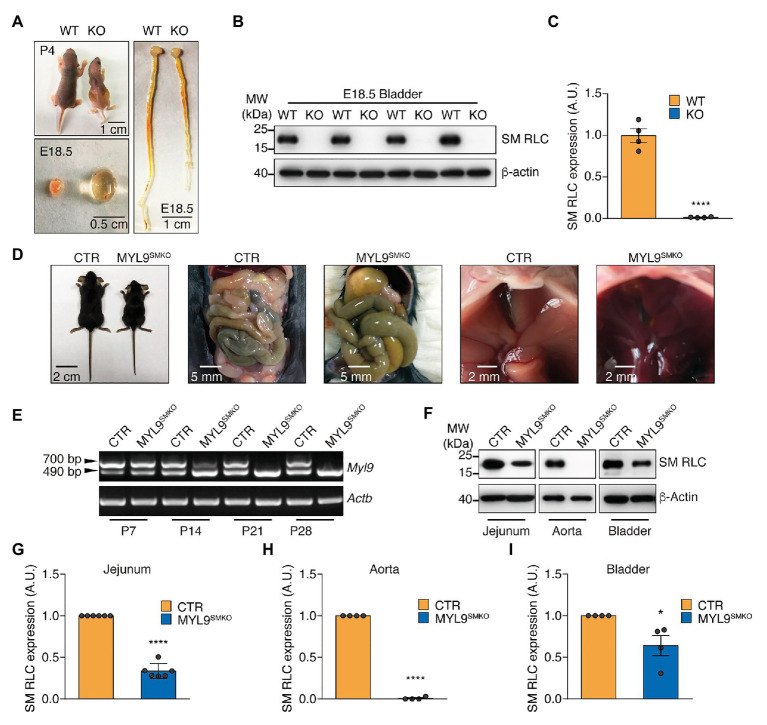
Characterization of *Myl9* knockout mice. **(A)** Photographs of wildtype (WT) and *Myl9^−/−^* (KO) mice at P4, as well as their bladder and gastrointestinal system at E18.5. The scale bars are labeled in the figures. **(B)** Western blot analysis of smooth muscle myosin regulatory light chain (SM RLC) expression levels in E18.5 KO and WT mouse bladder SM tissues. *β*-actin was employed as a loading control. **(C)** Statistical analysis of panel **(B)**. The values are expressed relative to that in WT. The data are presented as mean ± SEM (*n* = 4). ^****^p < 0.0001 (unpaired Student’s *t*-test, one-sided). **(D)** Photographs of control (CTR) and MYL9^SMKO^ mice at P28, as well as their gastrointestinal systems and livers. **(E)** Real-Time PCR (RT-PCR) detection of SM RLC mRNA levels in jejunum SM cells from CTR and MYL9^SMKO^ mice at different ages. The 700 bp band represents the wildtype allele, and the 490 bp band represents the knockout allele. **(F)** Western blot analysis of SM RLC expression levels at day P28 in jejunum, aorta, and bladder SM cells from CTR and MYL9^SMKO^ mice. β-actin was employed as a loading control. **(G–I)** Statistical analysis of panel **(F)**. The values are expressed relative to their loading control. The data are presented as mean ± SEM (jejunum: *n* = 6; aorta: *n* = 4; and bladder: *n* = 4). ^*^p < 0.05 and ^****^p < 0.0001 (paired Student’s *t*-test, one-sided). AU, arbitrary units; MW, molecular weight.

To determine the roles of SM and NM myosin II in hollow organs, we established a conditional knockout line on a C57BL/6 background; the *Myl9* gene was inserted with two *lox*P sites flanking exon 2 (Supplementary Figure S1A), and the resultant mice were crossed with different Cre lines. After crossing with the CMV-Cre line (Schwenk et al., 1995), we obtained a global *Myl9* knockout line. These KO mice (*Myl9*^−/−^) died within 1–4 days after birth (Figure 1A), but the number of offspring was in accordance with the Mendelian ratio. Macro phenotypes showed small body sizes, dilated bladders with a lot of urine, and dilated intestine lumens in the dead mice (Figure 1A). Western blotting showed no MYL9 protein expression in the mutant SM (Figures 1B,C), confirming complete deletion of the *Myl9* gene. This result indicated that global deletion of SM RLC led to severely impaired function in multiple hollow organs.

To avoid the lethal phenotype, we made a line with SM-specific deletion of the *Myl9* gene by crossing *Myl9^flox/flox^* mice with SMA (SM *α*-actin)-Cre mice (Wu et al., 2007). The resultant heterozygotes (*Myl9*^*flox*/+^; *SMA-Cre*) were used as CTR mice, while the homozygous (*Myl9*^*flox*/*flox*^; *SMA-Cre*, MYL9^SMKO^) mice were used as knockout mice. Similar to the CTR and WT mice, the knockout mice followed the expected Mendelian ratio for their births. At postnatal day 14 (P14), the knockout mice showed normal appearance, physiological behaviors, body weights and activities. At P21, the MYL9^SMKO^ mice had a similar body weight to the CTR mice (Supplementary Figure S1B). All the knockout mice died around P28, while the CTR mice survived to adulthood without any apparent phenotype (Supplementary Figure S1B). The knockout mice died with an enlarged abdomen caused by a dilated duodenum and jejunum containing abundant liquid (Figure 1D). To validate the knockout efficiency, we first detected the mRNA level of *Myl9* using a pair of primers targeting the 5'-UTR and 3'-UTR. The WT *Myl9* mRNA was still detected in jejunum SM tissues from 14-day-old MYL9^SMKO^ mice (Figure 1E), which was consistent with the normal phenotype. Western blotting analysis showed that Myl9 protein was not detected in aorta SM and was significant decreased but still detectable in the jejunum and bladder SM of P28 knockout mice (Figure 1F). The percentages of residual Myl9 protein in the mutant jejunum, aorta, and bladder SM were 33.56% ± 3.61, 0.78% ± 0.78, and 63.98% ± 12.13%, respectively (Figures 1G–I). Notably, it is reasonable that SMA-Cre led to different *Myl9* knockout efficiency in different SM tissues, since the expression of SMA in different SM tissues is regulated by various transcription factors and has distinct onset during development, for instance, it is regulated by Myocardin (*MYOCD*) in the airway SM cells, but not in vascular SM cells (Young et al., 2020). Histological analysis showed a dilated jejunum lumen with significantly increased SM layer thickness (Supplementary Figures S1C,F). The mutant liver appeared darker than the CTR liver (Figure 1D), and the mutant aorta SM layer became thinner (Supplementary Figures S1D,F), indicating vascular system impairment. There were no histological alterations in the mutant bladder (Supplementary Figures S1E,F), possibly due to the knockout inefficiency in the bladder SM. Taken together, the specific deletion of SM RLC in SM was sufficient to cause the functional impairment of hollow organs.

### SM RLC Is Required for SM Contraction

**Figure 2:**
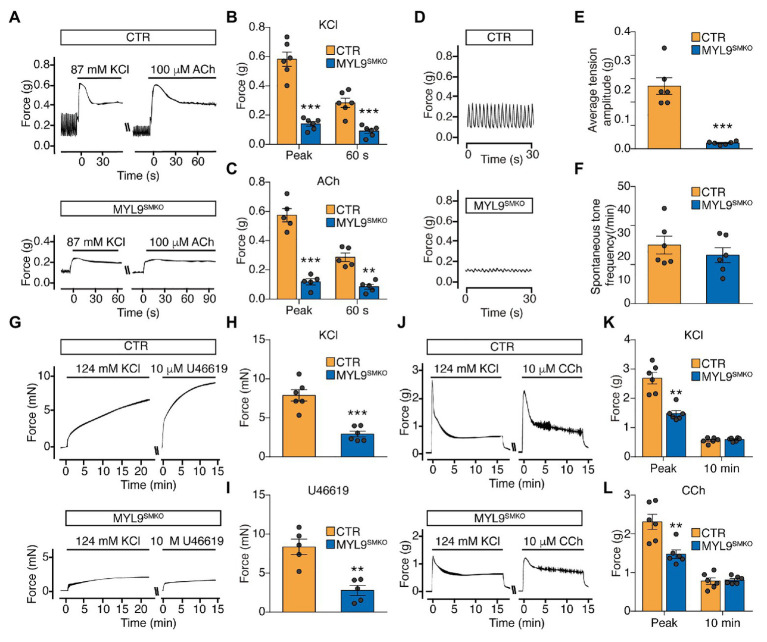
Smooth muscle contraction is impaired in SM-specific *Myl9* knockout mice. **(A)** Representative force tracing of the jejunum after stimulation with KCl and acetylcholine (ACh). **(B)** Statistical analysis of the KCl-induced force in panel (**A**; *n* = 6). **(C)** Statistical analysis of the ACh-induced force in panel (**A**; *n* = 5). **(D)** Representative peristaltic movement tracing of the jejunum. **(E)** Statistical analysis of the average tension amplitude in panel (**D**; *n* = 6). **(F)** Statistical analysis of the spontaneous tone frequency in panel (**D**; *n* = 6). **(G)** Representative force tracing of the aorta after stimulation with KCl and U46619. **(H)** Statistical analysis of the KCl-induced force in panel (**G**; *n* = 6). **(I)** Statistical analysis of the U46619-induced force in panel (**G**; *n* = 5). **(J)** Representative force tracing of the bladder after stimulation with KCl and CCh. **(K)** Statistical analysis of the KCl-induced force in panel (**J**; *n* = 6). **(L)** Statistical analysis of the CCh-induced force in panel (**J**; *n* = 6). The data are presented as mean ± SEM. ^**^p < 0.01 and ^***^p < 0.001 (paired Student’s *t*-test, one-sided).

The role of SM RLC phosphorylation in SM contraction has been well documented, but *in vivo* genetic evidence is currently lacking. We thus measured the tension of the *Myl9* SM-specific-deficient jejunum segment in response to KCl and the muscarinic agonist ACh. Upon stimulation with KCl, the jejunum from MYL9^SMKO^ mice showed a very weak initial contraction and sustained contraction, while the control muscle showed a typical strong initial contraction and rapid relaxation (Figures 2A,B). Quantitation showed that the initial force of the mutant jejunum SM was significantly smaller than that of the control (CTR: 0.582 ± 0.049 g vs. MYL9^SMKO^: 0.140 ± 0.016 g, *p* < 0.001). Upon stimulation with 100 μM ACh, contraction in the mutant jejunum SM was abolished at both the initial and sustained phases (Figures 2A,C). The maximal force tension was significantly smaller than that of the control jejunum (CTR: 0.574 ± 0.045 g vs. MYL9^SMKO^: 0.118 ± 0.021 g, *p* < 0.001). In addition, the peristaltic amplitude of the mutant jejunum muscle was significantly inhibited (Figures 2D,E), while the peristaltic frequencies were not altered (Figures 2D,F). Our results showed a required role for SM RLC in jejunum SM contraction. We also measured the contractile responses of aorta SM and obtained a similar result. The maximal forces evoked by depolarization (KCl) and agonist treatment (U46619) were both significantly decreased in KO muscle (Figures 2G–I). However, mutant bladder SM showed a significantly inhibited initial phase, whereas the sustained phase was comparable to that of the control (Figures 2J–L). Our results showed a required role for SM RLC in SM contraction but not sustained bladder contraction.

### SM RLC-Independent Contraction Is Mediated by NM Myosin

**Figure 3:**
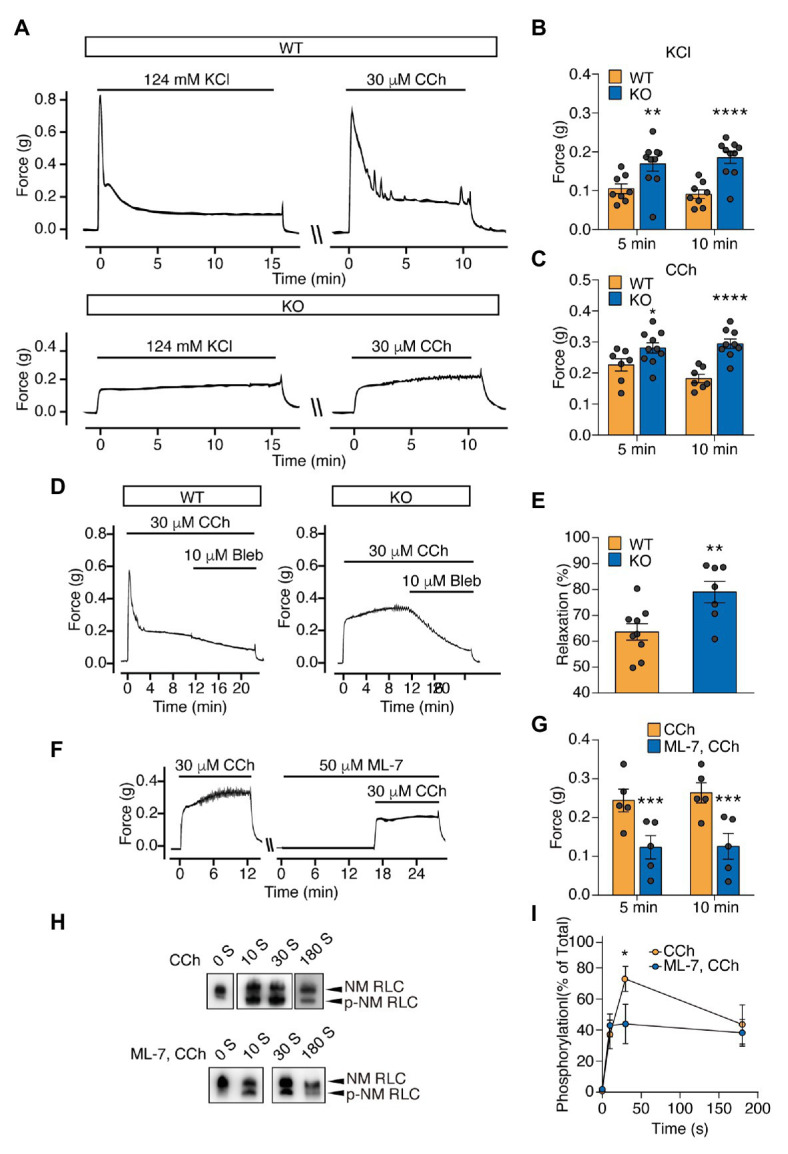
Sustained force is significantly increased in the bladder SM of *Myl9* global knockout mice at E18.5 and mediated by non-muscle (NM) RLC phosphorylation by myosin light chain kinase (MLCK). **(A)** Representative force tracing of bladder SM from E18.5 WT and KO mice. **(B)** Statistical analysis of the KCl-induced force at 5 and 10 min in panel (**A**; WT: *n* = 8; KO: *n* = 10). **(C)** Statistical analysis of the CCh-induced force at 5 and 10 min in panel (**A**; WT: *n* = 7; KO: *n* = 10). The data are presented as mean ± SEM. ^*^p < 0.05; ^**^p < 0.01 and ^****^p < 0.0001 (unpaired Student’s *t*-test, one-sided). **(D)** Representative force tracing of CCh-induced bladder SM contraction when the NM myosin inhibitor blebbistatin (Bleb) was applied. **(E)** Statistical analysis of the percentage of relaxation in Panel (**D**; WT: *n* = 9; KO: *n* = 7), Y value = the force after inhibitor application for 10 min/the force when Bleb was applied. The data are presented as mean ± SEM. ^**^p < 0.01 (unpaired Student’s *t*-test, one-sided). **(F)** Representative force tracing of E18.5 KO mice revealed bladder SM contraction before and after MLCK inhibitor (ML-7) treatment for 15 min. **(G)** Statistical analysis of the CCh induced force at 5 and 10 min, with or without ML-7 pretreatment in panel (**F**; WT: *n* = 5; KO: *n* = 5). The data are presented as the means ± SEM. ^***^p < 0.001 (paired Student’s *t*-test, one-sided). **(H)** Time-course of NM RLC phosphorylation in E18.5 KO bladder SM tissues induced by CCh with or without ML-7 application. The upper band and lower band represent non-phosphorylated and phosphorylated NM RLC, respectively. **(I)** Statistical analysis of the phosphorylated RLC level in panel (**H**; CCh: *n* = 5–7; ML-7, CCh: *n* = 4–6). The data are presented as mean ± SEM. ^*^p < 0.05 (unpaired Student’s *t*-test, one-sided).

To rule out the possible contribution of residual MYL9 protein to the sustained contraction of the *Myl9*^SMKO^ bladder, we measured the contractile response of E18.5 *Myl9*^−/−^ bladder SM with no MYL9 protein expression. Upon respective treatment with KCl and carbachol (CCh), the mutant muscle showed no initial peak force, which was consistent with the inhibited initial phase observed before. But to our surprise, the mutant muscle had a higher sustained force than the control (Figures 3A–C). These results strongly suggest SM RLC-independent contraction of bladder SM.

RhoA-associated kinase (ROCK) and its downstream target, the LIM domain kinase (LIMK), were reported to regulate RLC phosphorylation both directly and indirectly (Kureishi et al., 1997; Maekawa et al., 1999; Vicente-Manzanares et al., 2009). To investigate the contractile properties of the sustained contraction, we then applied the ROCK inhibitor H1152, LIMK inhibitor BMS-5 and the NM myosin II inhibitor blebbistatin (Bleb) to relax the evoked contraction of the *Myl9*^−/−^ bladder. Although both H1152 and BMS-5 relaxed mutant muscle, the extent was much smaller than that induced in control muscle (H1152: *p* < 0.05; BMS-5: *p* < 0.01; Supplementary Figure S2). In contrast, Bleb induced more profound relaxation in mutant muscle (WT: 63.61 ± 3.20% vs. KO: 79.02 ± 4.11%, *p* < 0.01; Figures 3D,E). This observation showed that the *Myl9*-independent contraction was sensitive to NM myosin II inhibition, implying the involvement of NM myosin II in SM contraction. As MLCK phosphorylates NM RLC to mediate contraction in SM cells (Kamm and Stull, 2001; Hong et al., 2011) and other cells (Holzapfel et al., 1983; Goeckeler and Wysolmerski, 1995) and regulate many other cellular processes, such as cell migration (Gallagher et al., 1997; Vicente-Manzanares et al., 2009), we also treated *Myl9*^−/−^ bladders with the MLCK inhibitor ML-7 15 min before CCh stimulation. We found that ML-7 dramatically decreased the force evoked by CCh (Figures 3F,G), suggesting that MLCK activation was required for the sustained force of the *Myl9*-deletion bladder.

### SM RLC-Independent Contraction Is Mediated by NM RLC Phosphorylation by MLCK

To determine if Myl9-independent contraction is mediated through NM RLC phosphorylation, we measured NM RLC phosphorylation of *Myl9*^−/−^ bladder SM with a urea/glycerol PAGE-based Western blot assay. Upon treatment with 30 μM CCh, KO bladder SM started to generate NM RLC phosphorylation at 10 s after stimulation, peaked (approximately 70% RLC phosphorylation) at 30 s, and then decreased gradually (Figures 3H,I). Note that NM RLC phosphorylation peaked much slower than SM RLC phosphorylation, which usually peaked within 10 s after stimulation (Chen et al., 2015), consistent with force development. To determine whether NM RLC phosphorylation is catalyzed by MLCK, we pretreated *Myl9*^−/−^ bladder SM with ML-7 and found significant NM RLC phosphorylation inhibition in response to stimulation (Figures 3H,I). This result indicated that NM RLC phosphorylation was catalyzed by MLCK during SM contraction.

### NM RLC Does Not Biochemically Compensate for SM RLC in SM

**Figure 4:**
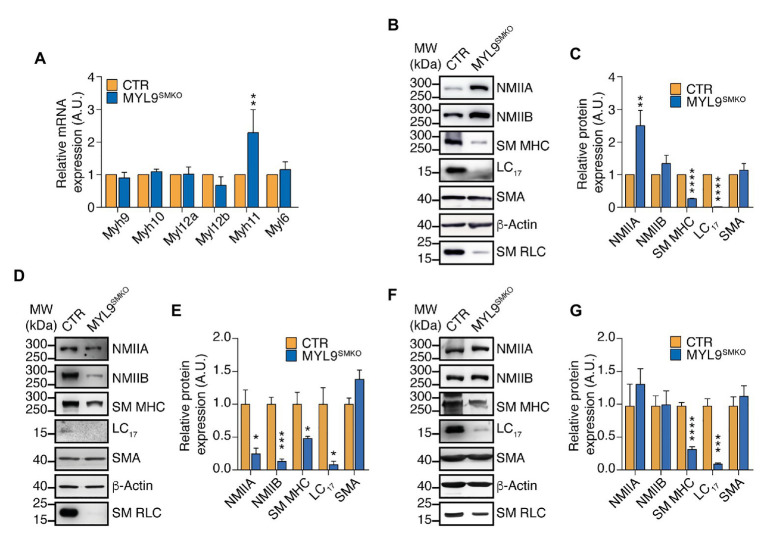
NM MHC is dramatically decreased in SM of *Myl9* conditional knockout mice. **(A)** Quantitative RT PCR analysis of SM and NM myosin component mRNA levels in the jejunum of CTR and MYL9^SMKO^ P28 mice (*n* = 4). **(B–G)** Western blot analysis of SM and NM myosin component protein expression levels in the jejunum **(B,C)**, aorta **(D,E)** and bladder **(F,G)** of CTR and MYL9^SMKO^ P28 mice. β-actin is employed as a loading control. **(C,E,G)** Statistical analysis of panel (**B,D,F**; *n* = 4). The values are expressed relative to their loading control. The data are presented as mean ± SEM. ^*^p < 0.05; ^**^p < 0.01; ^***^p < 0.001; and ^****^p < 0.0001 (paired Student’s *t*-test, one-sided). MW, molecular weight.

In light of the high sequence similarity shared by *Myl9*, *Myl12a*, and *Myl12b* encoded proteins, NM RLC-mediated contraction is expected to interact with SM RLC-mediated contraction through biochemical compensation. To determine the existence of this compensatory effect, we detected both the mRNA and protein levels of SM myosin II and NM myosin II components in P28 *Myl9*^SMKO^ jejunum SM. Quantitative real-time PCR analysis revealed that all the mRNA expression levels of the NM subunit genes, including two types of HC encoded genes (*Myh9* and *Myh10*), two types of RLC encoded genes (*Myl12a* and *Myl12b*) and an ELC encoded gene (*Myl6*), were unchanged, whereas the mRNA level of *Myh11*, a gene encoding SM MHC, was increased by two-fold in contrast to that in the control (Figure 4A). However, in mutant jejunum SM, NMIIA protein was significantly increased, while SM MHC protein was dramatically decreased (Figures 4B,C). SM myosin II and NM myosin II share the same ELC protein (Nabeshima et al., 1987), here, we almost cannot detect LC_17_ protein after *Myl9* deletion (Figures 4B,C), confirming that SM myosin II was almost depleted and suggesting that the content of NM myosin II was still very low. We also analyzed myosin protein levels in other SM tissues after *Myl9* was deleted. In both aorta and bladder, SM MHC and ELC proteins were significantly decreased, consistent with what was observed in jejunum. On the other hand, both NMIIA and NMIIB were decreased in aorta and were not changed in bladder (Figures 4D–G). Thus, this observation indicated that NM RLC unlikely assembled with the SM myosin II HC when SM RLC was absent, implying no compensatory effect of NM RLC.

### NM RLC-Mediated Contraction Is Prominent in the Uterus and Young Mice Bladder

**Figure 5:**
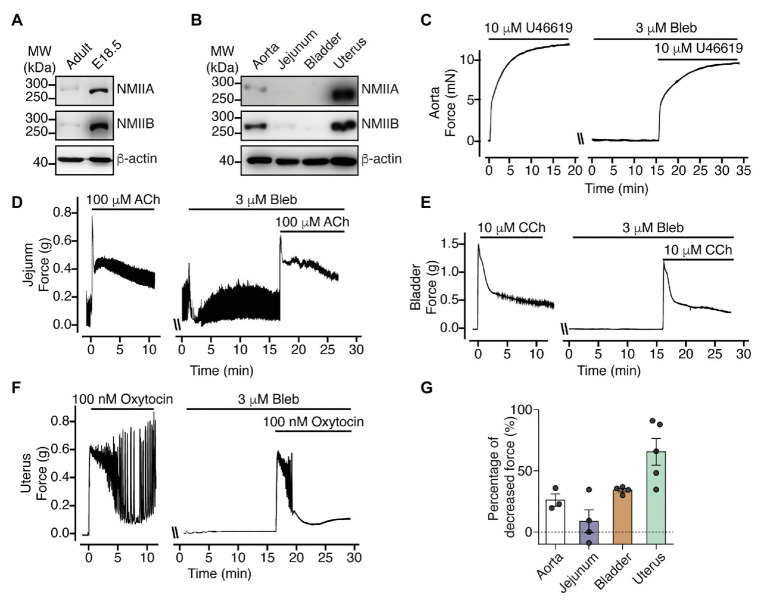
NM myosin II inhibition dramatically decrease uterine SM sustained contraction force. **(A)** Western blot analysis of NMIIA and NMIIB expression levels in bladder from adult and E18.5 mice. **(B)** Western blot analysis of NMIIA and NMIIB expression levels in aorta, jejunum, bladder and uterine SM from 8-week-old mice. **(C–F)** Representative force tracing of agonist-induced aorta **(C)**, jejunum **(D)**, bladder **(E),** and uterine **(F)** SM contraction with or without 3 μM Bleb pretreatment. **(G)** Statistical analysis of the percentage of decreased sustained force after pretreatment with Bleb in panels **(C–F)**. The data are presented as mean ± SEM (aorta: *n* = 3; jejunum: *n* = 4; bladder: *n* = 4; and uterus: *n* = 5). MW, molecular weight.

Because there are reports showing relatively high expression of NM myosin II HMC in the uterus and young mouse bladder compared to that in the aorta and stomach (Eddinger and Wolf, 1993), we expected that the relatively prominent force development may be mediated by NM myosin II. First, we measured the expression level of NMIIA and NMIIB in the bladder from E18.5‐ and 8-week-old mice and found that both NMIIA and NMIIB expression levels were much higher in the bladder from E18.5 than in the bladder from 8-week-old mice (Figure 5A). We also found that both NMIIA and NMIIB expression levels in uterus were dramatically higher than other SM tissues. Specifically, we almost cannot detect NMIIA and NMIIB signals in jejunum and bladder SM tissue (Figure 5B) under the same exposure condition. To test if the NM myosin II expression pattern is associated with NM RLC-mediated contraction, we then applied blebbistatin to force measurement of different SM tissues. Blebbistatin was reported capability to inhibit both SM and NM myosin II, but its efficacy with SM myosin II remains controversial (Limouze et al., 2004; Eddinger et al., 2007; Zhang et al., 2017). Here we used a relatively low concentration of blebbistatin in order to dissect the NM myosin II contribution to contraction in different SM tissues. Upon pretreatment with 3 μM blebbistatin, uterine SM from 8-week-old mice showed about 70% decrease in the oxytocin-induced sustained force, while the bladder, aorta and jejunum SM from 8-week-old mice all showed less than 40% decreased sustained force development (Figures 5C–G). These observations indicate that NM myosin II expression as well as NM RLC-mediated contraction is prominent in uterus and young mice bladder.

## Discussion

**Figure 6:**
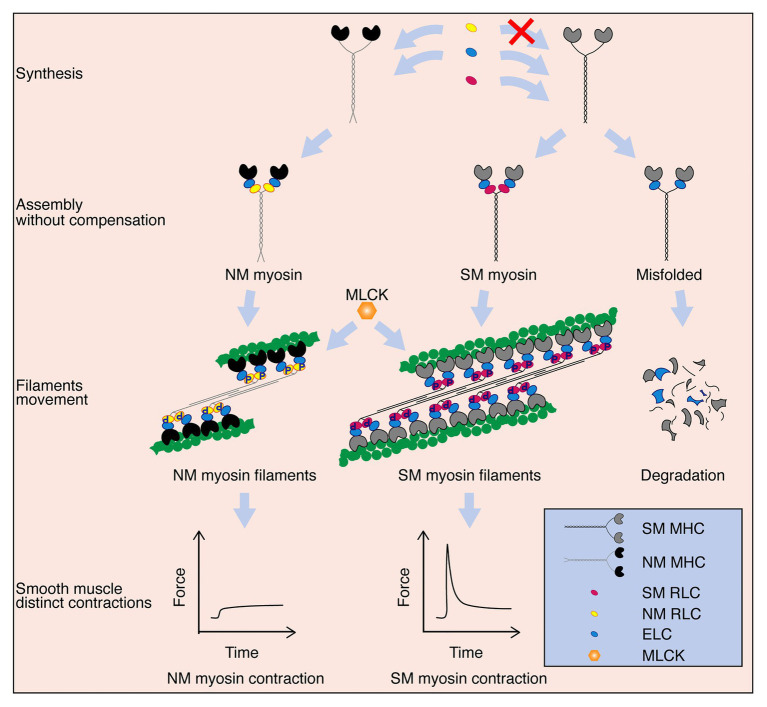
Schematic diagram of the proposed working model. SM myosin and NM myosin independently participate in regulating SM contraction. After these myosin components are synthesized, SM myosin and NM myosin are assembled. Both NM myosin and SM myosin are activated by MLCK mediated-RLC phosphorylation, which further lead to crossbridge movements between SM or NM myosin formed-thick and actin formed-thin filaments. NM myosin filament takes part only in the sustained phase of SM contraction, while SM myosin filament contributes to both the initial and sustained phases. Additionally, although NM myosin RLC and SM myosin RLC are extremely similar in their amino acid sequences and regulation ways, NM myosin RLC cannot compensate SM myosin RLC to assemble the complete and functional myosin. SM myosin will be misfolded and degraded eventually without RLC.

Hollow organs display diverse contractile behaviors, including peristalsis, tension maintenance and spasms, which are primarily conferred by different SM compositions. The SM myosin II and NM myosin II proteins within the SMs are usually co-expressed and thus potentially co-regulate contractile properties (Morano et al., 2000; Rhee et al., 2006; Ogut et al., 2007; Yuen et al., 2009). However, their distinct roles in SM contractility have not yet been determined. In this report, we found that SM-specific deletion of SM RLC dramatically decreased SM contraction and profoundly impaired hollow organ function. Global deletion of SM RLC can cause postnatal lethality in mice, with a similar phenotype as megacystis-microcolon-intestinal hypoperistalsis syndrome (MMIHS) patients who have homologous *MYL9* mutations (Moreno et al., 2018). This finding suggests a fundamental role of SM RLC in SM contractility and hollow organ physiological functions. In E18.5 global deletion SM RLC mice, we found that bladder SM still had a comparable sustained force, and this contraction was primarily due to MLCK-mediated phosphorylation of NM RLC (Figure 6). We thus suggest an essential role of NM RLC in SM contraction of young bladder and adult uterus, where expression of NM myosin is high. It was reported that NM myosin filaments mainly assembled at cortical compartment of the SM cells to regulate cortical actin polymerization and mediate force transmission (Zhang and Gunst, 2017). Combine with our data, we proposed that NM RLC did not biochemically and functionally compensate for SM RLC loss, the distinct roles appeared independent of each other (Figure 6). Under physiological conditions, although the ratio varies greatly with age and tissue origin, the amount of NM myosin II is much less than that of SM myosin II. When *Myl9* is deleted, structurally, NM RLC is unable to compensate for SM RLC to incorporate into SMHC. As a result, the incomplete SM myosin II molecule is disrupted (possibly *via* degradation) and hence SM myosin-mediated contraction is abolished. On the functional side, the NM myosin II is up-regulated after *Myl9* deletion to compensate for the inhibited force. Due to the variation of NM myosin II abundance, this compensation also varies. Identification of these distinct roles has important physiological implications for hollow organ physiology, e.g., in the bladder, abundant NM myosin II expression facilitates the production of the persistent force necessary for urine elimination, particularly for the young bladder when SM is not well developed. In the uterus, NM myosin II is highly expressed in the SM layer, which is useful for producing sufficient force to adapt the expanded uterus. In addition, it would be helpful to understand the alterative contraction properties of remodeling SM, particularly during abnormal NM myosin II expression in the vascular system.

A contracting action usually comprises an initial phase and a sustained phase. The prevalent view suggests that sustained contraction occurs primarily by a calcium sensitization mechanism (Somlyo and Himpens, 1989), which keeps RLC phosphorylation at a relatively high level and maintains the contraction through inhibition of the activity of myosin light chain phosphatase (MLCP) when the calcium concentration decreased in the sustained phase. The basic underlying mechanisms include constitutive phosphorylation of myosin phosphatase targeting subunit-1 (MYPT1; He et al., 2013; Chen et al., 2015) and extracellular signal induced activation of C-kinase-potentiated protein phosphatase 1 inhibitor of 17 kDa (CPI-17) *via* phosphorylated by protein kinase C (PKC; Kitazawa et al., 1999; Sun et al., 2019). Based on our observations in this report, sustained contraction mechanisms should include NM RLC-mediated contraction, at least in the bladder and uterus. However, we should note that NM RLC did not mediate calcium-sensitized contraction because it is dependent on MLCK activity, although its phosphorylation rate is relatively slow in contrast to SM RLC phosphorylation (He et al., 2008).

There are reports showing that NM RLC phosphorylation may be catalyzed by several kinases, including ROCK, ZIP kinase (ZIPK), and MLCK (Holzapfel et al., 1983; Cande and Ezzell, 1986; Goeckeler and Wysolmerski, 1995; Connell and Helfman, 2006; Vicente-Manzanares et al., 2009; Alcala et al., 2016). We found that MLCK inhibition significantly attenuated NM RLC phosphorylation as well as NM RLC-mediated force tension. This finding suggested that NM RLC phosphorylation was catalyzed by Ca/CaM-dependent MLCK during SM contraction. Notably, during other physiological processes or in NM cells, NM RLC phosphorylation may be catalyzed by kinases such as ZIPK rather than MLCK (Chen et al., 2014; Zhang et al., 2019).

It has been well documented that SM contraction is triggered by crossbridge movement of thin and thick filaments. However, NM myosin II does not incorporate into the thin/thick filaments, even in the absence of SM myosin II, and it mediates contraction possibly through other contractile mechanisms such as cytoskeleton reorganization (Zhang and Gunst, 2017; Zhang et al., 2018). However, we hypothesize that such cytoskeletal reorganization is different from cofilin-mediated cytoskeletal organization because the latter helps force transduction through the membrane and extracellular matrix rather than producing force autonomously. As NM RLC-mediated contraction is evolutionally conserved across species, the underlying contraction mechanism might be similar to that of the promyofibrils of muscular epithelial cells. However, the exact mechanistic processes remain to be determined.

## Data Availability Statement

The original contributions presented in the study are included in the article/Supplementary Material, further inquiries can be directed to the corresponding authors.

## Ethics Statement

The animal study was reviewed and approved by Institutional Animal Care and Use Committee of Model Animal Research Center of Nanjing University.

## Author Contributions

JS, TT, and M-SZ conceptualization and writing-review and editing. JS, Y-NQ, TT, WZ, L-SW, Y-QL, WW, YW, Y-YZ, XC, and X-NZ investigation. JS and TT visualization. JS, Y-NQ, and M-SZ methodology. JS and M-SZ writing-original draft. H-CP resources. M-SZ supervision and funding acquisition. All authors contributed to the article and approved the submitted version.

### Conflict of Interest

The authors declare that the research was conducted in the absence of any commercial or financial relationships that could be construed as a potential conflict of interest.
