# Supplementary material for: Distinct Roles of Smooth Muscle and Non-muscle Myosin Light Chain-Mediated Smooth Muscle Contraction
Source: Front Physiol. 2020 Dec 3;11:593966. doi: 10.3389/fphys.2020.593966 (PMC7793928; doi:10.3389/fphys.2020.593966)
Supplement: Supplementary file 1 [file Data_Sheet_1.pdf]

Supplementary Material

Supplementary Figures

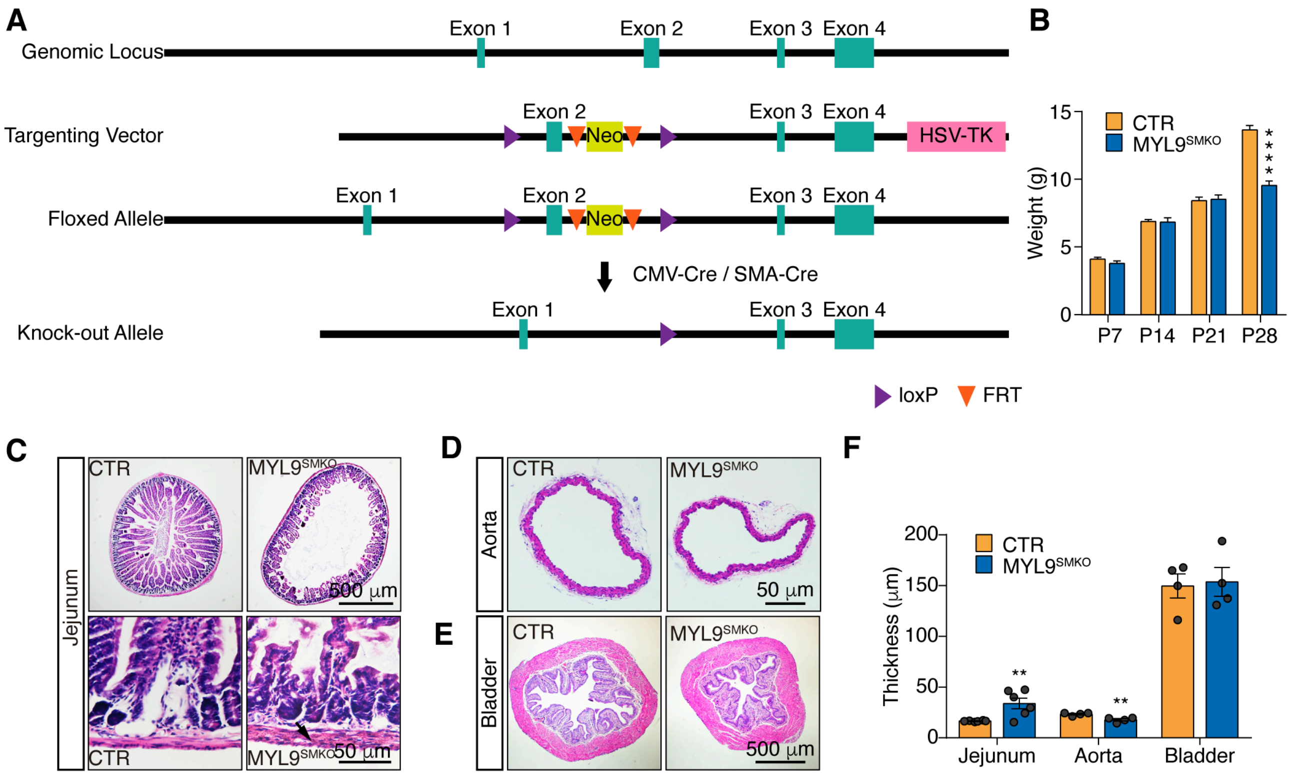

Supplementary Figure 1. Generation of *Myl9* knock out mice, and body weight and histological analysis of *Myl9* SM-specific-knockout mice. **(A)** Schematic representation of *Myl9* knock out strategy**.** Genomic DNA fragments containing *Myl9* gene were subcloned from BAC. A *lox*P site (purple triangle) and an *FRT*-Neo-*FRT*-*lox*P cassette were targeted to flank Exon 2. Mice containing the floxed allele were crossed with CMV-Cre or SMA-Cre to generate global knockout or smooth muscle specific knockout mice. **(B)** Body weights of CTR and MYL9^{SMKO} mice at P7, P14, P21 and P28. The data are presented as mean ± SEM (CTR: n = 20; MYL9^{SMKO}: n = 11). **** $P < 0.0001$ (unpaired Student's t-test, onr-sided). **(C-E)** Histological sections of jejunum **(C)**, aorta **(D)** and bladder **(E)** from CTR and MYL9^{SMKO} mice were stained with H&E. The scale bars are indicated in the figures. The arrowhead represents the MYL9^{SMKO} jejunum SM layer. **(F)** Statistical analysis of panel (C-E). The data are presented as mean ± SEM (Jejunum: n = 6; Aorta: n = 4; Bladder: n = 4). ** $P < 0.01$ (unpaired Student's t-test, one-sided).

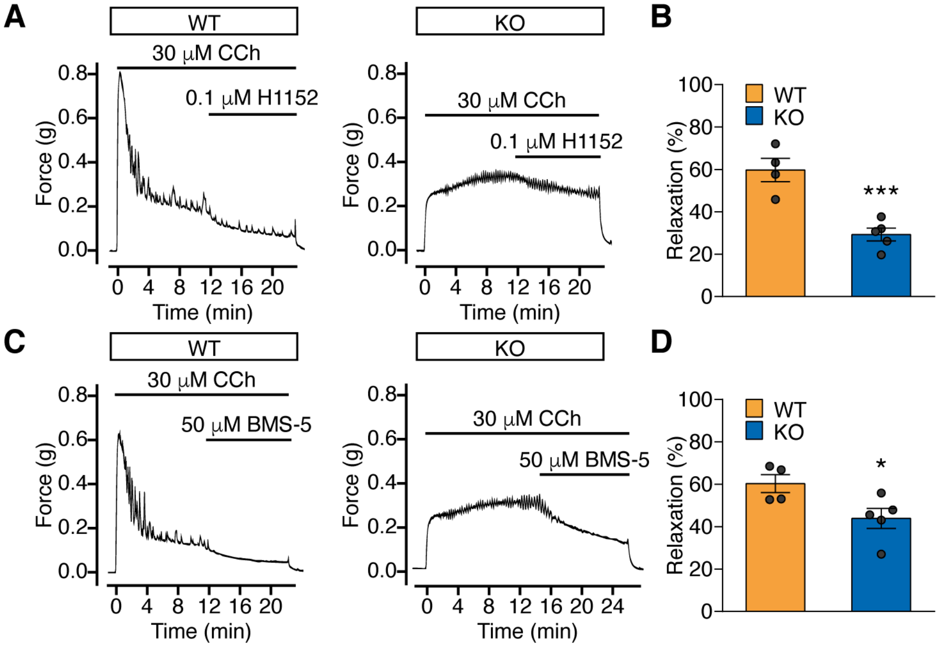

Supplementary Figure 2. H1152 and BMS-5 have less effects on relaxing mutant bladder SM than WT bladder SM strips. **(A)** Representative force tracing of CCh-induced bladder SM contraction when the ROCK inhibitor H1152 was applied. **(B)** Statistical analysis of the percentage of relaxation induced by H1152 in panel (a) (WT: $n = 4$; KO: $n = 5$). **(C)** Representative force tracing of CCh-induced bladder SM contraction when the LIMK inhibitor BMS-5 was applied. **(D)** Statistical analysis of the percentage of relaxation induced by BMS-5 in panel (C) (WT: $n = 4$; KO: $n = 5$). For panels (B) and (D), Y value = the force after inhibitor application for 10 min / the force when the inhibitor was applied. The data are presented as mean ± SEM. * $P < 0.05$; *** $P < 0.001$; (unpaired Student's t-test, one-sided).
